# HDAC4 as a potential therapeutic target in neurodegenerative diseases: a summary of recent achievements

**DOI:** 10.3389/fncel.2015.00042

**Published:** 2015-02-24

**Authors:** Michal Mielcarek, Daniel Zielonka, Alisia Carnemolla, Jerzy T. Marcinkowski, Fabien Guidez

**Affiliations:** ^1^Department of Medical and Molecular Genetics, King's College LondonLondon, UK; ^2^Department of Social Medicine, Poznan University of Medical SciencesPoznan, Poland; ^3^INSERM UMRS 1131, Université Paris Diderot, Institut Universitaire d'hématologie (IUH), Hôpital Saint-LouisParis, France

**Keywords:** histone deacetylase, signaling, HDAC4, neurodegeneration, HDAC inhibitors, therapeutic potential

## Abstract

For the past decade protein acetylation has been shown to be a crucial post-transcriptional modification involved in the regulation of protein functions. Histone acetyltransferases (HATs) mediate acetylation of histones which results in the nucleosomal relaxation associated with gene expression. The reverse reaction, histone deacetylation, is mediated by histone deacetylases (HDACs) leading to chromatin condensation followed by transcriptional repression. HDACs are divided into distinct classes: I, IIa, IIb, III, and IV, on the basis of size and sequence homology, as well as formation of distinct repressor complexes. Implications of HDACs in many diseases, such as cancer, heart failure, and neurodegeneration, have identified these molecules as unique and attractive therapeutic targets. The emergence of HDAC4 among the members of class IIa family as a major player in synaptic plasticity raises important questions about its functions in the brain. The characterization of HDAC4 specific substrates and molecular partners in the brain will not only provide a better understanding of HDAC4 biological functions but also might help to develop new therapeutic strategies to target numerous malignancies. In this review we highlight and summarize recent achievements in understanding the biological role of HDAC4 in neurodegenerative processes.

## Introduction

Transcription is a multistep process and its regulation involves a balanced coordination of several molecular factors. Epigenetic modifications of chromatin, including histone acetylation, represent priming events in the cascade leading to gene expression and are governed by the antagonistic activity of two families of enzymes: the histone acetyltransferases (HATs) and histone deacetylases (HDACs) (Fischer et al., 2010). The covalent modification of conserved lysine residues within histone proteins by acetyl groups leads to a nucleosomal relaxation and transcriptional activation; this reversible process provides a central mechanism to control gene expression and cellular signaling events. As such HDACs mediate epigenetic mechanisms that play a key role in homeostasis of histone functions and gene transcription. Mammalian HDACs are a family of 18 proteins divided into four groups based on structural and functional similarities: class I (HDACs: 1, 2, 3, 8), class IIa (HDACs: 4, 5, 7, 9), class IIb (HDACs: 6, 10), class III (sirtuins 1-7), and HDAC11 as the sole member of class IV (Saha and Pahan, 2006). It is well established that HDACs alter cell growth and differentiation by either governing chromatin structure or repressing the activity of specific transcription factors (Fischer et al., 2010). They are often deregulated in diseases and inhibition of their enzymatic activities remains of therapeutic interest.

Interestingly, the class IIa subgroup of HDACs shows a number of unique features in comparison to other HDACs (Verdin et al., 2003). Unlike the class I enzymes that are predominantly localized in the nucleus, class IIa HDACs shuttle between the nucleus and cytoplasm, a process that is controlled through the phosphorylation of specific serine residues (Chawla et al., 2003). The class IIa members are potent transcriptional repressors due to a high interaction propensity of N-terminal domains toward tissue specific transcription factors (Petrie et al., 2003; Saha and Pahan, 2006). Finally, the C-terminal catalytic domain of class IIa enzymes is characterized by a unique mutation of Tyr residue into His leading to the deacetylase domain inactivation (Lahm et al., 2007), emphasizing their distinctive properties compared to other HDACs (Verdin et al., 2003; Saha and Pahan, 2006).

HDAC4 shows approximately 60–70% sequence identity to HDAC5 and HDAC7 (de Ruijter et al., 2003). Among class IIa HDACs, HDAC4 has been described as a potent transcriptional repressor that is able to interact via its N-terminal domain with many different co-repressors, specifically in the brain. Hence, HDAC inhibitors have been recently used in the treatment of a wide-range of brain disorders characterized by pathological alterations in the transcriptome (Fischer et al., 2010) and they have displayed neuroprotective effects in animal models of such neurological disorders like Huntington's disease (HD), Alzheimer's disease (AD) and ischemic stroke.

## Regulation of HDAC4 expression, cellular localization and function

HDAC4 is ubiquitously expressed throughout the body with enrichment in the brain, heart and skeletal muscle (Verdin et al., 2003; Broide et al., 2007). However, the transcriptional mechanisms involved in the regulation of HDAC4 expression are poorly understood. Recent studies have shown that HDAC4 expression might be tightly regulated by microRNAs. In podocytes, miR-29a reduced HDAC4 signaling and attenuated the glucocorticoid-mediated β-catenin deacetylation and ubiquitination (Ko et al., 2013; Lin et al., 2014). Similarly, miR-365 reduced endogenous HDAC4 protein levels and led to inhibition of chondrocyte differentiation (Guan et al., 2011). Overexpression or inhibition of miR-206 in skeletal muscles has been associated with a decrease or increase of endogenous HDAC4 levels, respectively (Williams et al., 2009; Liu et al., 2012; Winbanks et al., 2013). HDAC4 expression has also been reported to be up-regulated under ER stress through its interaction with activating transcription factor 4 (ATF4). *In vitro*, HDAC4 overexpression caused ATF4 cytoplasmic retention and inhibition of ATF4 transcriptional activity, suggesting the presence of an autoregulatory loop. ER stress can ultimately promote cell apoptosis through up-regulation of ATF4 target genes such as CHOP and TRB3 and this effect was exacerbated by HDAC4 down-regulation (Zhang et al., 2014).

**Figure 1:**
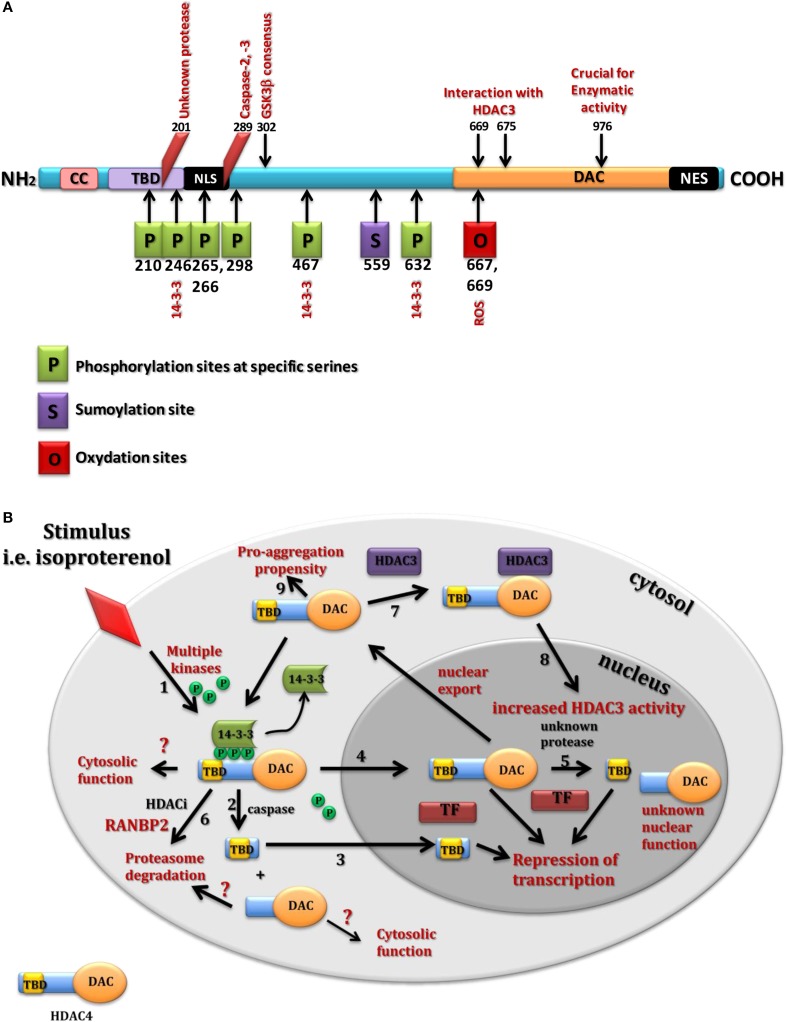
**Structure and cellular function of HDAC4**. **(A)** Summary of HDAC4 post translational modifications. CC, coil-coil domain; TBD, transcription binding domain; NLS, nuclear localisation signal; NES, nuclear export signal; DAC, deacetylation domain. **(B)** Summary underlying HDAC4 cellular localization and identified functions spanning all tested systems. HDAC4 undergoes nuclear-cytoplasmic shuttling in response to different stimuli through multiple kinases (1). In the cytoplasm, HDAC4 might be cleaved by proteases (2) to generate a small N-terminal fragment that translocates into the nuclei (3) to bind different transcription factors (TF) and repress their driven transcription. Similarly the full length HDAC4 upon dephosphorylation by phosphatases (4) translocates into the nuclei to act as a repressor of TF. In the nuclei, HDAC4 is cleaved by unknown protease to produce a distinct nuclear N-terminal fragment (5). Treatment with HDACIs might lead to the RANBP2-mediated proteasome degradation of HDAC4 (6). HDAC4 as a non-active deacetylase can also bind HDAC3 (7) to enhance its deacetylase activity (8). HDAC4 as a scaffolding protein prompts to form many complexes and has showed a cytosolic pro-aggregation propensity in HD mouse models (9).

It is believed that HDAC4 undergoes a signal-dependent shuttling between the cytoplasm and nucleus, although in the brain it seems to be exclusively localized to the cytoplasm (Darcy et al., 2010; Mielcarek et al., 2013a,b). This nuclear-cytoplasmic shuttle is controlled by multiple mechanisms including activity of calcium/calmodulin-dependent kinase (CaMK) (Bolger and Yao, 2005) and salt-inducible kinases (Walkinshaw et al., 2013a), cAMP signaling (Walkinshaw et al., 2013b), and oxidative stress (Matsushima et al., 2013) (reviewed in Parra and Verdin, 2010, Figure 1A). Under normal conditions phosphorylated HDAC4 was retained in the cytoplasm through its association with 14-3-3 proteins (Grozinger and Schreiber, 2000; Verdin et al., 2003), (Figure 1A) and once dephosphorylated at Ser298 by the catalytic subunit of PP2A (Protein Phosphatase 2) it moved into the nucleus (Paroni et al., 2008).

It has been shown that HDAC4 can produce three specific nuclear pools including full length HDAC4 and two N-terminal fragments with different functions controlling cell death and differentiation *in vitro* (Paroni et al., 2004, 2007; Backs et al., 2011). Indeed, HDAC4 protein can be cleaved by caspases leading to a HDAC4-nuclear fragment generation (Paroni et al., 2004, 2007). Cleavage of HDAC4 occurred at Asp289 and resulted in the formation of a cytosolic carboxy-terminal fragment and an amino-terminal fragment that accumulated into the nucleus. This nuclear fragment exhibited a stronger cell death-promoting activity coupled with increased repressive effect on Runx2 (Runt-related transcription factor 2) or SRF (Serum response factor) dependent transcription. Interestingly, this nuclear fragment was a less potent inhibitor of MEF2C (Myocyte enhance factor 2C)-driven transcription, compared to full-length HDAC4 (Paroni et al., 2004), although such repressor activity has been described as independent from the acetylase domain. While caspase-2 and caspase-3 have been shown to cleave HDAC4 *in vitro*, caspase-3 was critical for HDAC4 cleavage *in vivo* during UV-induced apoptosis (Paroni et al., 2004). In the nucleus, a caspase-generated HDAC4 fragment was also reported to trigger cytochrome C release from mitochondria and cell death in a caspase-9-dependent manner (Liu and Schneider, 2013). In isolated skeletal muscle fibers expressing a HDAC4-green fluorescent protein, activation of PKA by the beta-receptor agonist isoproterenol or dibutyryl cAMP caused a steady HDAC4 nuclear influx. Thus, mutations of Ser265 and Ser266 (PKA targeted serines) enabled HDAC4 to respond to PKA activation (Liu and Schneider, 2013). Similarly, clenbuterol a potent β_2_-adrenoreceptor stimulator in skeletal muscles caused HDAC4 phosphorylation on Ser246 through activation of CaMKII (Ohnuki et al., 2014). In cardiomyocytes, PKA induced generation of the N-terminal HDAC4 cleavage product at Tyr202. This N-terminal fragment selectively inhibits activity of MEF2 but not SRF, thereby antagonizing a pro-hypertrophic potential of CaMKII signaling without affecting cardiomyocytes survival. Thus, HDAC4 may function as a molecular nexus for the antagonistic actions of the CaMKII and PKA pathways (Backs et al., 2011). In addition, sustained glycolysis induced by lipopolysaccharide (LPS) treatment activated caspase-3, which cleaved HDAC4 and triggered its degradation. Importantly, a caspase-3 resistant HDAC4 mutant escaped LPS-induced degradation and prolonged inflammatory cytokine production through the GSK3β (Glycogen Synthase Kinase-3 β isoform)–iNOS (inducible Nitric Oxide Synthase)–NO (Nitric Oxide) axis (Wang et al., 2014a). However, until now, there have been no data available suggesting a similar proteolytic pattern of HDAC4 in the healthy brain or in neurodegenerative disorders.

Interestingly, cleavage and phosphorylation sites are all located within the N-terminal region of HDAC4 protein highlighting this area as an important regulatory domain. While this N-terminal region seems to be critical for the repressive function of HDAC4, it also contains a transcription factor interacting domain that can bind MEF2 family members. HDAC4-MEF2 interaction was associated with the inhibition of MEF2 function resulting in neuronal cell death (Mao et al., 1999) and repression of MEF2-dependent genes in neuronal cells (Bolger and Yao, 2005) and skeletal muscles (Miska et al., 2001). In addition, the HDAC4 N-terminal region is characterized by a high glutamine content that is likely responsible for interactions with other glutamine-rich proteins leading to a spontaneous assembly of insoluble toxic amyloid-like structures (Fiumara et al., 2010). X-ray resolution of the human HDAC4 glutamine-rich domain showed that this domain is preferentially folding into a straight alpha-helix which assembles into a tetramer. In contrast to the coiled coil proteins, the HDAC4 tetramer lacked the regular arrangement of apolar residues and had an extended hydrophobic core that might lead to its rapid equilibrium with monomer and intermediate species (Guo et al., 2007). Overall, these studies provide a picture of a multifunctional protein and emphasize the presence of several mechanisms behind the tissue-specific regulation of HDAC4 expression and function.

## Regulation of HDAC4 deacetylase activity

Previous findings have suggested that class IIa HDACs are inactive on acetylated substrates, thus differing from class I and IIb enzymes (de Ruijter et al., 2003). HDAC4 catalytic domain purified from bacteria was 1000-fold less active than class I HDACs on standard substrates (Lahm et al., 2007). In contrast to the other HDACs, the C-terminal catalytic domain of the class IIa enzymes contains an amino acid substitution of a critical Tyr residue into His (Lahm et al., 2007). Mutation of this Tyr to His in class I HDACs severely reduced their activity, while a His-976-Tyr mutation in HDAC4 produced an enzyme with a 1000-fold higher catalytic efficiency (Lahm et al., 2007). Interestingly, mutations in the residues involved either in the coordination of the structural zinc binding domain of HDAC4 or the binding site of class IIa selective inhibitors prevented the association of HDAC4 with N-CoR/HDAC3 associated repressor complex (Bottomley et al., 2008). It has been proposed that HDAC4 binds directly to HDAC3 in order to activate its deacetylase domain (Mihaylova et al., 2011) and that the structural zinc-binding domain is crucial in the regulation of class IIa HDAC functions (Bottomley et al., 2008).

Finally, HDAC4 activity seems to be modulated by the ubiquitin-proteasome system. Serum starvation elicited the polyubiquitination and degradation of HDAC4 in non-transformed cells. Phosphorylation of Ser298 within the PEST1 sequence, a GSK3β consensus sequence, played an important role in the control of HDAC4 stability. Phosphorylation of HDAC4 by GSK3β has been described to occur *in vitro* upon phosphorylation of Ser302, which seems to play a role of a priming phosphate (Cernotta et al., 2011) and removal of growth factors fails to trigger HDAC4 degradation in cells deficient in this kinase (Figure 1B). One might conclude that HDAC4 is not a histone/protein deacetylase, however it can play a crucial role in many processes through its interaction with HDAC3 or with a general role of scaffolding protein.

## HDAC4 biological function in non CNS organs

As mentioned, HDAC4 is ubiquitously expressed, however, its initial biological function was described in chondrocytes: direct inhibition of RUNX2 by HDAC4 led to chondrocyte hypertrophy (Vega et al., 2004). HDAC4-null mice displayed premature ossification of developing bones due to an ectopic and early onset chondrocyte hypertrophy, mimicking the phenotype associated with the constitutive *Runx2* expression in chondrocytes. On the other hand, overexpression of HDAC4 in proliferating chondrocytes *in vivo* inhibited their hypertrophy and differentiation, mimicking a *Runx2* loss-of-function phenotype (Vega et al., 2004).

HDAC4 has been described as a critical factor that connects neural activity to the muscle remodeling program, nevertheless, its role in the physiology of this peripheral tissue is controversial and has not been entirely clarified. Inactivation of HDAC4 suppressed denervation-induced muscle atrophy while increased re-innervation (Williams et al., 2009; Winbanks et al., 2013). Although it has been observed that miR-206 could regulate HDAC4 expression in skeletal muscles, the postnatal expression of miR-206 is not a key regulator of a basal skeletal muscles mass or specific pathways of muscle growth and wasting (Winbanks et al., 2013). Previous studies established that muscle denervation strongly induced the expression of Gadd45a, a small myonuclear protein that is required for denervation-induced muscle fiber atrophy. Interestingly, it was shown that HDAC4 mediated an induction of Gadd45a mRNA in denervated skeletal muscles (Bongers et al., 2013). Furthermore, HDAC4 has been described to induce AP1-dependent transcription by activating the HDAC4-MAPK-AP1 signaling axis essential for the neurogenic muscles atrophy (Choi et al., 2012). Interestingly, AP1 inactivation recapitulates HDAC4 deficiency and blunts the muscle's atrophy program. Surprisingly, HDAC4 stimulated AP1 activity by activating the HDAC4-MAPK-AP1 signaling axis essential for the neurogenic skeletal muscles atrophy (Choi et al., 2012). HDAC4 was also described as a member of the MEF2C repressor complex together with HDAC3 and Ser/Thr kinase homeodomain-interacting protein kinase 2 (HIPK2) in undifferentiated myoblasts (de la Vega et al., 2013). On the other hand, a recent study has shown that HDAC4 inactivation led to defective satellite cells proliferation, muscle regeneration and lipid accumulation (Choi et al., 2014).

Finally, it was shown that HDAC4 up-regulation was significantly greater in patients with rapidly progressive ALS (Amyotrophic lateral sclerosis) and its expression was negatively correlated with a degree of skeletal muscles re-innervation and functional outcome (Bruneteau et al., 2013). Similarly an increased level of HDAC4 has been found in SMA (Spinal muscular atrophy) model mice and in SMA patient muscles (Bricceno et al., 2012). Moreover, HDAC4 expression was increased in masseter muscles from a patient with a deepbite and was found to correlate negatively with slow myosin type I and positively with fast myosin type IIX (Huh et al., 2013). Overall, these studies provide evidence of an active role of HDAC4 in the neurogenic muscle's atrophy program, which becomes exacerbated in some neurodegenerative disorders, therefore, urging HDAC4 as a promising therapeutic target.

## HDAC4 function in the brain and its implication in neurodegeneration

Compared to the other class IIa enzymes, HDAC4 is highly expressed in the mouse brain (Grozinger et al., 1999; Darcy et al., 2010) with a highest expression occurring during early postnatal life (Sando et al., 2012). Immunohistochemical analysis of brain sections revealed accumulation of HDAC4 in the cytoplasm of neurons, including neurons containing CRH, oxytocin, vasopressin, orexin, histamine, serotonin, and noradrenaline (Takase et al., 2013). HDAC4-immunoreactive puncta were uniform in size and were widely distributed in the neuropil of brain areas, including the PVN (Hypothalamic Paraventricular Nucleus), LHA (Lateral Hypothalamic Area), ARC (Hypothalamic Arcuate Nucleus), TMN (Tuberomammillary Nucleus), DR (Dorsal Raphe), and LC (Locus Coeruleus). Interestingly, these HDAC4 positive accumulations co-localized with PSD95-immunoreactive puncta (Mielcarek et al., 2013a; Takase et al., 2013), suggesting a role for HDAC4 in synaptic plasticity (Sando et al., 2012; Mielcarek et al., 2013a).

In neurons, dynamic changes in the subcellular localization of HDACs are thought to contribute to various signaling pathways. Treatment with the neuronal survival factor BDNF (Brain-derived neurotrophic factor) suppressed HDAC4 nuclear translocation, whereas a pro-apoptotic CaMK inhibitor stimulated HDAC4 nuclear accumulation. Moreover, as expected, an ectopic expression of the nuclear-localized HDAC4 led to neuronal apoptosis and repressed the transcriptional activities of survival factors in neurons like: MEF2 and cAMP response element-binding protein (CREB). In contrast, inactivation of HDAC4 by small interfering RNA or HDAC inhibitors suppressed neuronal cell death (Bolger and Yao, 2005). In cultured hippocampal neurons, localization of HDAC4 has been shown to be very dynamic and signal-regulated and spontaneous electrical activity was sufficient for HDAC4 nuclear export (Chawla et al., 2003). On the other hand, in various experimental models, it has been shown that loss of HDAC4 could lead to neurodegeneration of the retina (Chen and Cepko, 2009) and cerebellum (Majdzadeh et al., 2008). This might be explained by the chondrocyte hypertrophy that occurred in mice lacking HDAC4 causing developmental brain abnormalities due to a skull deformation (Vega et al., 2004) and might be further supported by major pathological changes in the *Hdac4* knock-out murine postnatal brain (Majdzadeh et al., 2008). In addition, a conditional knock-out of *Hdac4* under the *CamkII* promoter in the mouse forebrain, showed impairments in the hippocampal-dependent learning and memory with a simultaneous increase in locomotor activity (Kim et al., 2012). However, it was recently shown that a selective deletion of *Hdac4* under the control of the *Thy1* or Nestin promoters resulted in a normal gross brain morphology and cytoarchitecture as well in a normal locomotor activity (Price et al., 2013). Moreover, the Affymetrix array data showed no effect of *Hdac4* knock-out on the transcriptional profile and global acetylation of the postnatal murine brain (Mielcarek et al., 2013b). Similarly, a hippocampal depletion of HDAC4 *in vivo* abolished long-lasting stress-inducible behavioral changes and improved stress related learning and memory impairments in mice (Sailaja et al., 2012). HDAC4 overexpression has been shown to accelerate the death of cerebellar granule neurons (Bolger and Yao, 2005; Li et al., 2012; Sando et al., 2012) by increasing their vulnerability to H_2_0_2_ insult due to an inhibition of PPARα activity (peroxisome proliferators-activated receptor α) (Yang et al., 2011). In addition, the HDAC4 viral-mediated overexpression in the rat hippocampus was sufficient to induce depression like behavior (Sarkar et al., 2014). Interestingly, overexpression of HDAC4 in the adult mushroom body, an important structure for memory formation, resulted in a specific impairment in long-term courtship memory but had no affect on short-term memory in *Drosophila* model (Fitzsimons et al., 2013). Similarly, HDAC4 and HDAC5 increased a cell viability through an inhibition of HMGB1, a central mediator of tissue damage following acute injury and it has been shown that NADPH oxidase-mediated HDAC4 and HDAC5 expression contributed to the cerebral ischemia injury through the HMGB1 signaling pathway that could be an effective therapeutic target to treat stroke (He et al., 2013).

## HDAC inhibitors and inhibition of HDAC4 activity

Suberoylanilide hydroxamic acid, known also as SAHA or vorinostat, was the first HDAC inhibitor (HDACI) to be approved for the cancer therapy of advanced cutaneous T-cell lymphoma (Marks and Breslow, 2007). Initially, SAHA was identified as an inhibitor of class I and class II HDACs at nanomolar concentrations (Richon et al., 1998), but was further characterized as inhibitor of class I HDACs as well specifically HDAC6 within the class IIb enzyme (Parmigiani et al., 2008; Marks and Xu, 2009). More recently activity based probes have been used to demonstrate that SAHA can bind directly to both class I and IIa HDACs (Salisbury and Cravatt, 2007; Codd et al., 2009). Experiments performed on cancer cell lines revealed the ability of SAHA to induce the degradation via RANBP2-mediated proteasome of both HDAC4 and HDAC5 *in vitro* (Scognamiglio et al., 2008). Interestingly, other HDACIs, such as trichostatin A (TSA) and sodium butyrate, have also been reported to induce a reduction in HDAC4 levels when administered to embryoid bodies (Chen et al., 2011), suggesting that similarly to SAHA, these other HDACIs could induce HDAC4 degradation through a proteasome-dependent mechanism.

An increased expression of HDAC4 has been described in several *in* vitro and *in* vivo models of neuro-like disorders. Treatment of neuronal cell lines with SAHA led to a noticeable improvement of cell polarity and morphology, with longer processes in the rat H19-7 hippocampal cell line with folate deficiency. In this neuronal cell model, folate deficit led to a reduction in cell proliferation and decreased production of S-adenosylmethionine (the universal substrate for transmethylation reactions) concurrent with an increased expression of HDACs (including HDAC4,6,7) (Akchiche et al., 2012). Furthermore, cecal ligation and peroration (CLP) rats, used in the Sepsis-associated encephalopathy (SAE) study, were also characterized by an increased expression of HDAC4 (Fang et al., 2014). Administration of HDACIs (e.g., TSA or SAHA) restored Bcl-X_L_ and Bax levels *in vivo* and decreased apoptotic cells *in vitro*. In addition, knock-down of HDAC4 by shRNA resulted in an enhanced histone acetylation like: H3 and H4 and reduced neuronal apoptosis. Consistently, CLP rats treated with TSA or SAHA exhibited significant spatial learning and memory deficits with no effect on their locomotive activity (Fang et al., 2014).

In preclinical settings, SAHA and other HDACIs have consistently improved the phenotype in HD mouse models (Ferrante et al., 2003; Hockly et al., 2003; Gardian et al., 2005) and are being developed as HD therapeutics. Recent findings have increasingly described a widespread peripheral organ pathology in HD, such as skeletal muscles atrophy (Zielonka et al., 2014a) and heart failure (Mielcarek et al., 2014a; Zielonka et al., 2014b), often associated with an increased HDAC4 expression (Mielcarek et al., 2014b). As such, class IIa HDACs inhibitors might be beneficial in delaying HD-related symptoms and, therefore, are under evaluation as HD therapeutics. It has been shown that the administration of SAHA to wild type and R6/2 mice decreased HDAC2 and HDAC4 at the protein but not RNA levels in different brain regions *in vivo* (Mielcarek et al., 2011), supporting previous observations from cancer cell lines (Scognamiglio et al., 2008). We have also shown that HDAC4 associates with huntingtin in a polyglutamine-length dependent manner and co-localizes with cytoplasmic inclusions. Consequently, reduction of HDAC4 levels delayed cytoplasmic aggregate formation in different brain regions of R6/2 mice and rescued cortico-striatal neuronal synaptic function in HD mouse models. This was accompanied by an improvement in motor co-ordination, neurological phenotypes and increased lifespan (Mielcarek et al., 2013a). Interestingly, SAHA treatment of R6/2 mice was accompanied by restoration of brain-derived neurotrophic factor (BDNF) cortical transcript levels (Mielcarek et al., 2011). An increased expression of BDNF has been associated with memory-enhancing and neuroprotective properties of HDACIs, as it has been shown that HDAC4 and HDAC5 might repress specific *Bdnf* transcripts in rats and primary hippocampal neuronal cultures and this effect was reversed by SAHA treatment (Koppel and Timmusk, 2013). However, the mechanism of BDNF induction by HDACIs is not yet fully understood. Surprisingly, HDAC4 reduction had no effect on global transcriptional dysfunction and did not modulate nuclear huntingtin aggregation in HD mousse models (Mielcarek et al., 2013a). Interestingly, elevated HDAC4 levels have been shown in *post mortem* HD (Yeh et al., 2013) and FTLD (Frontotemporal Lobar Degeneration) (Whitehouse et al., 2014) brains and HDAC4 has been described as a component of Lewy Bodies in Parkinson's disease brains (Takahashi-Fujigasaki and Fujigasaki, 2006) and of intranuclear inclusions in the neuronal intranuclear inclusion disease (Takahashi-Fujigasaki et al., 2006). Consistently, administration of SAHA has been shown to improve synaptic plasticity and learning behavior in an Alzheimer disease model (Kilgore et al., 2010). A causative role for HDAC4 has been also described in SCA-1 (Spinocerebellar Ataxia Type 1) as a modulator of Ataxin-1. It was shown that ataxin-1 bound specifically to HDAC4 and MEF2 and co-localized with them in the nuclear inclusion bodies. Significantly, these interactions were greatly reduced by the S776A mutation, which largely abrogates the cytotoxicity of ataxin-1 *in vitro* (Bolger et al., 2007). Moreover, HDAC4 has been found to be significantly overexpressed in specific cortical regions of autistic patients (Nardone et al., 2014).

Diabetes is one of major risk factors for dementia. However, the molecular mechanism underlying the risk of diabetes for dementia is largely unknown. Surprisingly, it has been shown that diabetes may cause epigenetic changes in the brain, which adversely affect synaptic function. These alterations were associated with an increased susceptibility to oligomeric Aβ-induced synaptic impairments in the hippocampal structure that eventually led to synaptic dysfunction. Use of pharmacological inhibitors against the HDAC IIa family restored synaptic function (Wang et al., 2014b). This therapeutic effect highlights the importance of HDACIIa members, including HDAC4, as a possible target in the brain.

## Conclusion

**Figure 2:**
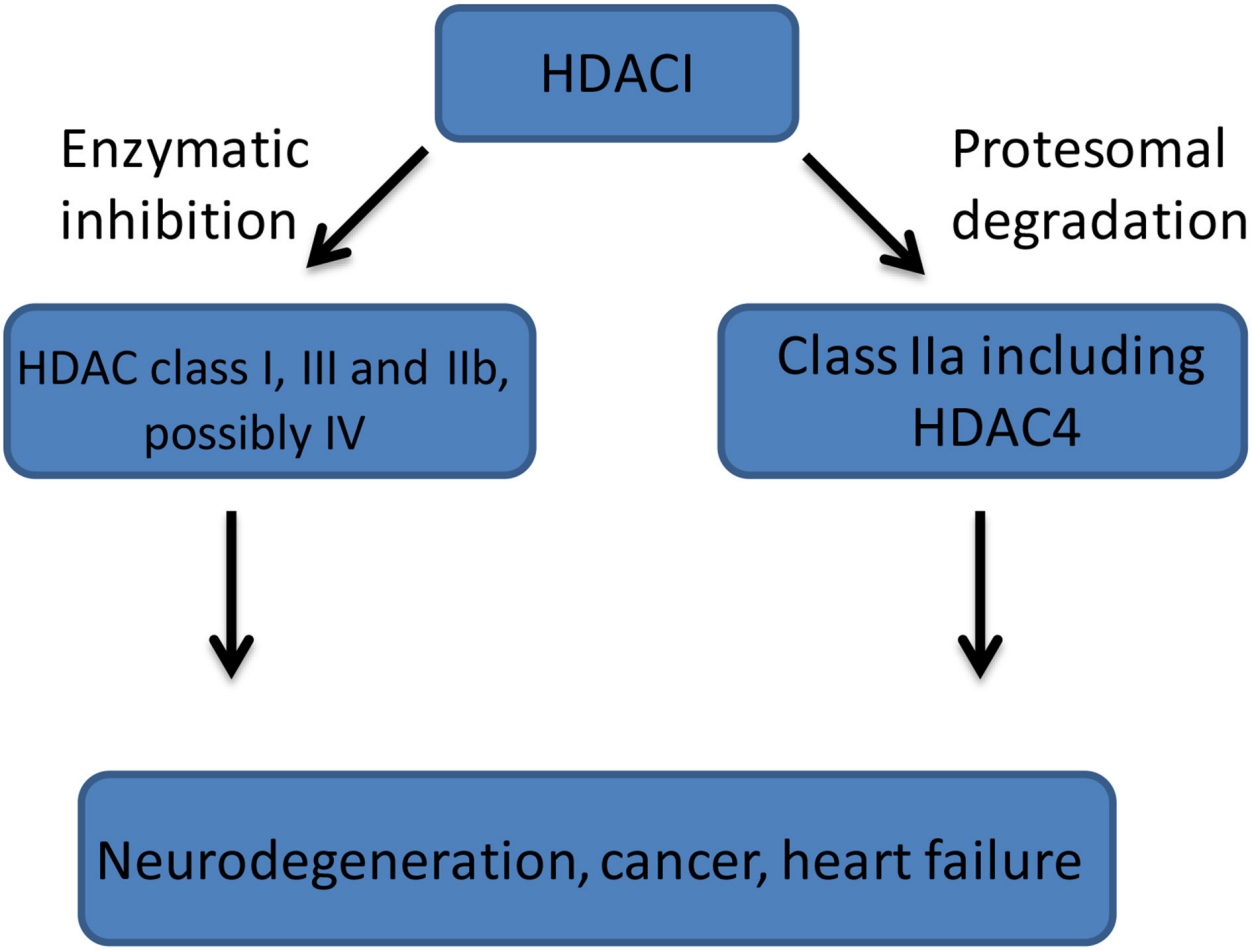
**A general mechanism of HDACIs (HDAC Inhibitors) action**. Class I (HDACs: 1, 2, 3, 8), Class IIa (HDACs: 4, 5, 7, 9), Class IIb (HDACs: 6, 10), Class III (sirtuins 1-7), and Class IV (HDAC11).

There is convincing evidence suggesting that HDAC4 plays a central role in the brain physiology and that it is deregulated in several neurodegenerative disorders, therefore representing a suitable therapeutic target, through which HDACs inhibition may occur. However, the use of currently available HDACIs is likely to involve adverse side effects due to the broad spectrum HDAC inhibition. Therefore, a search for selective HDAC inhibitors would likely be of benefit for targeted therapy. Likely, HDACs inhibition occurs through a dual mechanism, either by a direct inhibition of an active deacetylase domain (class I, IIb, III, and IV) or by a direct binding followed by proteosomal degradation (class IIa) (Figure 2). Crucially, relatively little is known regarding individual HDACs functions in the adult brain. Although, class I HDACs biological functions have been intensively studied in the brain, it appears that suitable HDAC targets could arise from HDAC IIa subfamily but their function and role are still poorly understood. Recent studies identified HDAC4 as a critical component of several neurological processes including neuronal survival and synaptic plasticity in healthy and diseased brains. However, little is known about HDAC4 cellular process like: mechanisms governing HDAC4 cellular localization, post-translational modification and a proteolytic cleavage, especially in the diseased brains. In addition, HDAC4 transcriptional regulation has not been studied and therefore the description of the specific transcription factors and regulatory elements driving HDAC4 expression should be carefully undertaken. The presence of an inactive deacetylase domain within the class IIa HDACs might also suggest that newly designed small molecules should be rather directed to the HDAC4 known functions, including transcription binding domain, HDAC3 interaction or proteolytic cleavage sites than toward deacetylase domain. Therefore, much more research is needed to fully describe biological function of HDAC4 in the healthy and diseased brains to be able to shape future therapeutic strategies for a various disorders.

### Conflict of interest statement

The authors declare that the research was conducted in the absence of any commercial or financial relationships that could be construed as a potential conflict of interest.
